# Fathers in the Care of Children with Disabilities: An Exploratory Qualitative Study

**DOI:** 10.3390/healthcare10010014

**Published:** 2021-12-22

**Authors:** Brenda M. Uribe-Morales, Pablo A. Cantero-Garlito, Carmen Cipriano-Crespo

**Affiliations:** Faculty of Health Sciences, University of Castilla La Mancha, 45600 Talavera de la Reina, Spain; breenmoo1999@outlook.com (B.M.U.-M.); MariaCarmen.Cipriano@uclm.es (C.C.-C.)

**Keywords:** caregiving, fathers, caregivers, care of others, quality of life, children with disabilities, occupational therapy

## Abstract

Objective: to explore the perception and experience of fathers of children with disabilities in caring for their children; to know their role and how these tasks impact their daily life, health and physical, mental and/or emotional well-being. Methodology: qualitative approach study with phenomenological design. The sample consisted of seven young fathers of underage children with various diagnoses. The data were collected through semi-structured interviews; the discourse analysis was carried out through open and axial coding processes. Three themes emerged from the results of the analysis: (1) shared responsibilities, (2) somewhat difficult to fit in, and (3) either you join or you split. Results: Fathers must readjust their work schedule, reduce their working hours, or give up their job altogether to take care of their children, as well as give up their social life. They lack time to enjoy their leisure time, to spend time with their partner, to take care of themselves. This involvement in caregiving generates an important occupational imbalance that has repercussions above all on their mental health. Conclusions: The sharing of caregiving tasks also impacts significantly on parents’ lives, it also takes away time and opportunities, and sometimes health and quality of life.

## 1. Introduction

To speak of care implies understanding all those tasks necessary to ensure the daily well-being of all people, tasks that are present throughout the life cycle in one form or another. For this reason, it is necessary to understand the concept as a fundamental attribute for the maintenance and sustainability of life, and as a basis for the development of social life [1]. Although the term includes countless activities (childbirth, child rearing, feeding, supervision, listening, cooking...), it is a recent term and generally understood as “those tasks that are done to maintain the well-being of people in a situation of dependency, that is, who cannot look after themselves because of some disability” [2]. However, as Joan Tronto and Berenice Fisher point out, caring is “an activity characteristic of the human species that includes everything we do to maintain, continue or repair our “world” so that we can live in it as well as possible. This world includes our bodies, our individualities, and our environment, which we try to maintain in a complex web that sustains life” [3].

In any case, care in general, and child-rearing more specifically, have traditionally been tasks associated with the role of women in any culture and context [4]. As a result, there is still an unequal distribution of care tasks between women and men, as they are not usually understood as a male responsibility. Thus, unpaid care work is mostly carried out by women in their homes, and paid care work continues to be feminized, less valued and poorly paid (nursing assistants, household cleaners, caregivers, etc.) [5]. This unequal distribution of care tasks has a significant impact on women’s lives, as it takes away time and opportunities, and in many cases health and quality of life [6]. Traditionally, studies on caregiving have focused more on the tasks carried out by women [7,8]. However, in recent years, greater attention has been paid to caregiving undertaken by men. Recent studies shows an increase in their involvement in caregiving, as well as a rethinking of the priorities and functions they carry out [9,10]. It would therefore be worthwhile to look more closely at how caregiving is carried out when men are responsible for the care of children with disabilities.

The reality is no different for mothers whose children suffer from a disability or chronic health problem; on the contrary, care tasks can even intensify and lengthen over time, as activities such as dressing, feeding or personal hygiene can become a daily task throughout the child’s life. This is confirmed by numerous studies: mothers are the main caregivers of children with disabilities and sometimes end up abandoning their jobs to cope with this work, and those who also work are subjected to double the demands and overload, as many of the paid work they do is also linked to the field of care [5,11,12,13].

There are many studies [5,6,13,14,15] that confirm the feminization model of caregiving and address the impact of caregiving on women’s lives, but very few that focus on the role of fathers in this family dynamic and in the care of children with special needs. Those that do address it show that the care tasks in which they are involved are generally different from those of women, since they tend to dedicate themselves mainly to the economic support of the household, and do not participate in the care routines focused on grooming, showering, dressing and feeding; on the contrary, they participate in more satisfying, less repetitive and stressful activities: leisure time, games and entertainment, walks outside the home, or occasional supervision while the mother performs other care tasks, such as cooking, laundry or cleaning [12,16]. A study [17] conducted in the United States comparing 6572 fathers and 7376 mothers suggests that fathers’ attachment to caregiving is largely dependent on employment and income, so they are more likely to be involved in routine caregiving when their wives contribute a significant portion of the household income. Other studies [4,5,16,17] suggest that women’s increasing entry into the labor market has not always been accompanied by a proportional increase in men’s participation in caregiving occupations, again overburdening the mother or other women in the family. In any case, the results of the study [17] show that fathers spend much less time with children than mothers, approximately 31 h per week (50 h/s for the mother).

For parents the birth of a child is a source of stress but, according to the data found in a systematic review [18], it seems that when the child has a health problem this stress intensifies notably more in mothers than in fathers, they tend to experience depressive periods and emotional anguish that persists over time as a consequence of the need for constant care (and this differentiates them from mothers of children without disabilities). Men, on the other hand, experience greater stress due to the impact of child care on marital life and marital intimacy, referring to not spending time as a couple without the child and even abandoning the family more frequently [16]. Their concerns also include the social acceptance of the child and their own attachment to them. For others, however, income is the most worrying issue.

When coming to terms with the arrival of a child with health problems, while mothers experience more sadness, fathers feel more rage and anger [18] and show feelings of inadequacy related to guilt [19], they may blame the mother for the “biological failure” or feel guilty for not having provided their children with the necessary components to lead a healthy and happy life [16]. They seek social and emotional support from other family members and are more likely to express their emotions publicly than mothers are. Fathers are more likely to avoid or deny the problem, hiding their vulnerability, not accepting that their child is different and delegating the responsibility of care, etc. Disappointment is not something present in the parents. However, they often admit to comparing their children with other children of their age, their expectations are usually related to them managing to develop basic skills to be self-sufficient [16].

In terms of the use of health and social services for the treatment of their children, it is the mothers who are the main providers; they find it less difficult to interact with health professionals than the fathers, as they do not seem to have immediate needs, they do not interact and therefore are often perceived negatively by professionals who seem to have assumed that, in terms of care, the mother is the only provider or the person to whom they should turn [18]. In the same way, Rivard’s study [19] found that fathers of children with genetic disorders felt similar to mere observers of their children, ignored by both their wife and the professional on most occasions.

On the other hand, mothers of children with disabilities compared to mothers of children without health problems experience a more significant deterioration in their physical health. This translates into muscle aches, high levels of anxiety and stress, exhaustion, physical fatigue, burnout, feelings of intense anger, guilt, depression.

However, occupational therapy has barely addressed the occupational performance of fathers of children with disabilities in relation to care tasks, and even less so for men, even though from our field of competence it is necessary to understand how caring for a child with a disability alters, hinders, and impacts the lives of families, and not only of mothers. Knowledge of the division of care tasks will help occupational therapists to address the needs of these children in the family context, as well as those of their parents. Some authors [5,11,12] agree on the need to delve deeper into this subject as a future line of research. The lack of information on the subject justifies the desire to carry out this study, whose main objective will be to explore men’s perception and experience of caregiving tasks.

The general objective of this study is to explore the perception and experience those fathers of children with disabilities have of caring for their children. More specifically, it aims to describe and analyze the way in which men are involved in caregiving tasks, specifically those related to taking care of children with disabilities and dependency, and to explore the impact of the birth of a child with disabilities on the daily life of parents and the family system, as well as on their physical, mental and/or emotional health and wellbeing.

## 2. Materials and Methods

### 2.1. Study Design

The present study was carried out using a qualitative methodology and from a phenomenological approach [20]. This approach was used because it is based on the study of life experiences from the perspective of the subject [21,22,23]. The phenomenological design allows the description of phenomena through the narrative of the subjects who experience it, explaining the way they live it and interpret it. In this way, we were able to explore, describe and understand the meaning that the participants gave to their reality and their experience as fathers of a child with a disability.

### 2.2. Participants

The selection of participants was carried out during the months of March, April, and May 2021. The following inclusion criteria were considered for their participation in the study: (1) being the parent of a child with a disability, (2) residing in the same family home as the minor, and (3) voluntarily participating in the study by signing the informed consent form. Exclusion criteria: fathers whose children are 18 years of age or older and/or who do not live with the minor.

The recruitment strategy consisted of first contacting various associations and special education schools in Toledo and Talavera de la Reina, hoping that these institutions could put us in contact with those fathers who met the inclusion criteria and who wanted to participate voluntarily in the study. In this way, only two participants were obtained. Finally, we used social media recruitment, snowball sampling and word of mouth.

The sample finally obtained consisted of seven fathers of minor children with various diagnoses (hearing impairment, 1P36 deletion syndrome, Down syndrome, Mowat Wilson syndrome, ADHD accompanied by high abilities and oppositional defiant disorder, and ASD) and from different Spanish cities (León, Talavera de la Reina, Alicante, Madrid, Toledo). Table 1 shows some of the main characteristics of the participants.

Interested fathers were contacted by telephone to explain to them in detail the objective of the study and to arrange an appointment at their convenience for an in-depth interview. They were provided with the information about the study and the informed consent form by e-mail, which those who were interested had to sign. It was indicated that participation was completely voluntary and anonymous, as it was reiterated that the information handled would be confidential.

### 2.3. Data Collection

The collection of information was carried out between April and May 2021. The technique used was the semi-structured interview guided by a series of open-ended questions (Supplement Table S1) to encourage the narrative of the participants. These were conducted with the seven participants by telephone, face-to-face was not contemplated considering the limitations caused by COVID-19 as well as the location of some participants. All interviews were audio-recorded and transcribed verbatim; they ranged in length from 15 to 50 min. To ensure anonymity, all personal characteristics of the participants that could facilitate their identification were removed from this article. Once the interviews were transcribed, the original audios were destroyed. These interviews were conducted by the researcher himself.

### 2.4. Data Analysis

The recordings were subsequently transcribed with the help of Journalist Studio software and analyzed with MAXQDA 2020 software (VERBI Software GmbH, Berlin, Germany). Data analysis began with reading and re-reading the transcripts. The participants’ accounts were analyzed thematically; these were initially coded following an inductive approach, without pre-established analysis criteria or hypotheses. The different members of the research team met regularly throughout the duration of the study to analyze and review the data obtained.

The discourse analysis process was carried out according to the following sequence. Once the units of analysis were established, an open coding process was carried out, conducted independently by two researchers, following a constant comparison procedure [22]. Thus, 171 data codes were established, which were subsequently grouped into 23 subcategories. Then, through an axial coding process, the subcategories were integrated into broader categories. Subsequently, the codes were reduced to themes after an inductive process involving three researchers from the team where the codes and themes were discussed. Finally, the categories were grouped into three themes, corresponding to the objectives of the study, in which the participants’ experiences and meanings about the phenomenon studied are accurately captured.

### 2.5. Ethical Considerations

This study was approved by the Clinical Research Ethics Committee of the Integrated Health Management Area of Talavera de la Reina (Code 8/18). This research has complied with legislation related to data protection, in particular the General Data Protection Regulation of the European Union (EU Regulation 2016/679 of 27 April) and Organic Law 3/2018 of 5 December on Data Protection and Guarantees of Digital Rights. The EU Charter of Fundamental Rights and Law 14/2007, of 3 July, on biomedical research (BOE, 4 July 2007) have also been considered. Likewise, the entire study has been carried out in accordance with the Ethical Standards and Recommendations of the Declaration of Helsinki.

## 3. Results

The findings from the analysis of the interviews have resulted in three main themes describing how fathers are involved in caring for their children with disabilities and the impact of caring on their daily lives: (1) Shared responsibilities; (2) Something difficult to fit in; (3) Either you separate or you join in. Each of the above categories is explained in detail below and illustrated with quotes from the participants.

### 3.1. Shared Responsibilities

For the men who participated in the study, caring for their children with disabilities is a shared responsibility, especially when their children’s daily difficulties require constant care:

*“She doesn’t respond to her environment, she can’t stand up, she can sit up a bit, but we can’t say that she has good head control... they ended up placing a gastric button, so she eats through her belly, we give her food with a syringe because she doesn’t eat through her mouth. She is totally dependent, man, babies are dependent in themselves, but she is more dependent even at the age she is”*.

(P3)

Although everyone is involved in different ways in the care of their children depending on the time, they have available, fathers admit to participating in basic care tasks, those related to the personal autonomy of their children, and divide these tasks with their wives. Showering and grooming, feeding, dressing, preparing meals, taking medication and even physical therapy at home are tasks in which fathers participate daily to a greater or lesser extent, thus relieving the overload on mothers.

*“... the medication it’s usually me who gives it to her, the food, although my wife prepares it, I usually give it to her, although not always every meal but most of the time I do; and I also do a little bit of physical therapy with her...”*.

(P3)

Mothers in this case do not assume the role of sole caregivers, some of them occupy the figure of main caregiver so that most of their time is spent caring for their children and the tasks then become their responsibility. On the other hand, those who do not spend the whole day at home, the time they do spend at home is usually dedicated to these care routines (showering, feeding, and dressing).

Going to medical appointments, check-ups, rehabilitation centers and/or associations is also a regular task for these fathers, either alone or accompanied by their wives.

*“We usually both go to therapy... I can tell you that if 19–20 people attend on average there have been many sessions that there has only been one father and that was me”*.

(P6)

The need for constant care of their children cannot always be met by fathers, as their involvement always depends on the time their jobs allow them to spend at home. In fact, this was one of the central themes in their narratives. For all, having a child with special needs at home has in one way or another affected their jobs, having to adapt and reduce their working hours or give up work.

*“Since then, I have a part-time, reduced working day and so I only work during the time he is at school and then I can look after him in the afternoons. My wife has a full day”*.

(P6)

This is not something that only affects fathers, since mothers in this case are the ones who have mostly had to leave their jobs to take care of their children, and for those who were not yet working *“the doors of opportunities that they could have chosen were closed to them”* (P7).

*“Before, we both worked... my wife has taken a leave of absence and she is the one who takes care of him”*.

(P5)

Not being able to spend as much time at home as they would want to is a concern for fathers, who feel the need to share caregiving duties to ease the burden on their wives:

*“I would like to be able to spend more time at home, for us to be in a different situation and for me to be the one to ask for leave and my wife to work, or for both of us to be able to work and share the heavy burden of looking after a child like that, but it’s not possible now”*.

(P5)

They mention that deciding how and who adapts their working day has been an important decision, it is usually something that has been contemplated as a couple. It is hardly something that she alone decides, between the two of them they decide what is best based on several issues: “ease” to ask for the reduction of the working day or leave, whoever finds a job first (when they do not have one), proximity between work and home, or economic issues. In no case was it assumed that the responsibility was exclusively the woman’s, the decision was a compromise between the two.

*“We simply evaluated other types of things, first of all who had the better salary so that when it came time to reduce, it was the one who earned the least who reduced and above all the one who generated the least disruption”*.

(P6)

In any case, the time dedicated by one or the other is not always equal, it depends above all on work, since in no case did the fathers interviewed mention any other issue as a limitation for taking care of their children full time. For those fathers who are not full-time carers, the mornings before school, evenings after 7 o’clock and weekends are the times when they spend the most time with their children.

*“During the week maybe when I leave work until bedtime, from 7 to 9:30 maybe. But I spend more time practically at the weekend because my work doesn’t allow me to do so”*.

(P5)

The fathers do not perceive differences between the care provided by the mother and the care provided by them, admitting that their needs with them are covered without any difference. The only thing to highlight is that they tend to show more affection, that they tend to be more affectionate with their children, and that they spend more time with them daily than they do:

*“She spends twice or three times as much time as I do, but when I take care of him, I think we take care of him in a very similar way (...) in general I don’t think she can take care of him better than I can if I have that time”*.

(P5)

### 3.2. Something Difficult to Fit

All described the news of their children’s disability as devastating, difficult to cope with, a time filled with fears, worries about their children’s future, and uncertainty.

*“Well, it was a blow, a difficult period, I am still in psychological treatment for it, and well, it is something difficult to fit in, it is something that is going to be for life and that does not have a solution as such”*.

(P2)

They recognize that in front of others they were strong, and admit that the hard blow really came later, when you are aware of the limitations of your child, or when after the expectations he/she does not understand what you expect. One of the fathers points out:

*“There in the consultation you hold your nerve in a sense, but it’s difficult is difficult because you hear the one word you don’t want”*.

(P7)

When facing parenthood with standards and expectations, if your child has a disability that will limit his or her entire life is even more complicated. Fathers mention that they always end up expecting their children to go further than expected, or comparing their children to other children their age, which causes frustration and prolongs the pain over time.

*“... honestly there are days when I take it worse and in general, I take it well, but there are days when I also have my downs because it is a blow, it is a reality, because where is the child, the son that you longed for, and also the temptation of the comparison leaks in”*.

(P7)

All fathers remember the process of acceptance as a hard time, sometimes more so for the mother. However, emotionally it was also hard for them to take, and they admit to breaking down often:

*“The mother suffered more. Even today it is more difficult for her than for me”*.

(P3)

*“The truth is that I don’t remember how she handled it... but I remember my process with a lot of anguish or bitterness”*.

(P7)

This internal struggle between what you expect and what you have is not only present in the first few months. After the diagnosis of their children, the daily care, and the sacrifices consequently, the health and well-being of these fathers has also been affected. One parent points out:

*“If there is any parent with a child with a disability that doesn’t have an impact on their physical and emotional health, I would want to meet them”*.

(P7)

In this case all the fathers mentioned suffering more emotional than physical damage, even stating that they are no longer the same as they were before; they are more tired, depressed, frustrated and nervous this is how they describe themselves when we ask them how the diagnosis of their children has impacted their health, even though for some of them the news was received more than 10 years ago. This is due, according to the fathers, to the fact that the daily difficulties are more complicated to assume than the news itself, which for some was even reassuring after a long process of study.

*“For a long time I was quite down, now thank goodness I am not, but it is as if something inside me had turned off (...) it is not really due to taking care of my son because the one who takes care of him most of the time is not me, but it is due to knowing that my son is not and can never be what I expected as a father”*.

(P5)

However, the demands also activate them, help them to be alert and committed to their children’s needs:

*“...what I’m trying to do now is to do more exercises with weights at home, kettlebells, dumbbells.... Why? Because I see that it is necessary to pick her up”*.

(P3)

### 3.3. Either You Separate or You Join

Caring for a child with a disability implies, in addition to work sacrifices and physical and emotional problems, that fathers give up other satisfying activities such as leisure and free time. Taking care of another person on a daily basis means that fathers pay little attention to their own care, leaving aside enjoyment with their partner, with friends, and satisfying activities in general. Life changes suddenly, for some in a very negative way, as they say: “I consider that my life is now worse than before” (P2). However, it is not something they attribute directly to having a child with a disability, they all mention that life changes when you have a child:

*“... if he were not a dependent person, now, with more age, he would have more autonomy and that would entail for us more autonomy and freedom to resume activities that we may have put aside or to share more as a couple”*.

(P5)

In this way, the couple’s relationship also changes. It is a reality that everyone emphasizes. For those who were already close before the birth of their child, this new stage contributes to that union. For others, however, the added stress deteriorates it:

*“It has had the same negative impact, in other words, my relationship with my wife is not good at the moment”*.

(P2)

In addition to this, the absence of free time makes it difficult to “do things as a couple”, being alone and talking about other topics that do not have to do with the children is almost impossible. Vacations, weekend outings, going to the movies, dinner, are things that require time, and they say they don’t have it. This fact is attributed to the reality of their children because it is difficult for them to imagine how it would have changed if it had been otherwise.

*“It’s true that I would like to be able to go away for a weekend alone, but damn it’s impossible, how can you give yourself a break and overload someone else with your burdens”*.

(P5)

If they don’t have time to spend with their partner, they don’t have time for themselves. They acknowledge having given up their hobbies, spending time with friends, and sports since they became fathers; however, it is not something they have difficulty with, on the contrary, they are happy with it because they understand that this is what must happen, and when you are the parent of a child with a disability this is more complex:

*“We had to each do our part, give up our self, our personal ambitions, which had already been frustrated, for the common good or for the good of others. In this case, for me the common good is her and my son, and for her it is me and her son (...) if there are sacrifices when you are a parent, then when you have a child with a disability, they are multiplied by three”*.

(P7)

In this way, those fathers involved in the upbringing of their children with disabilities also suffer the consequences of loss of freedom and time.

## 4. Discussion

The present study aimed to explore the perceptions and experiences of fathers of children with disabilities. For the fathers who participated in this study, the birth of their children with disabilities and being involved in their care has had a significant impact on different areas of their lives: many of them have had to readjust their working hours, reduce their working day or give up their job altogether to care for their children; they have given up their social life, they lack leisure time, time to spend with their partner, to do sport or rest, to take care of themselves. These consequences have also increased the already existing stress and caused a decrease in the emotional well-being of these fathers. So, the distribution of care tasks also has a significant impact on men’s lives, it also takes away time and opportunities, and sometimes health and quality of life. As other studies [19] have shown, caring for a child with a disability has significant consequences for fathers’ physical, mental, and social well-being, freedom and independence, family well-being and financial stability.

Mothers in our research are not the only caregivers or those who suffer the consequences of providing constant care [4,5,6], some of the fathers are the main caregivers, so they spend most of their time caring for their children, which allows them to be involved in routine care tasks: they shower their children, dress them, feed them, prepare food, among other activities that relieve the overload of their wives. This does not follow what we found in other studies [5,13,18] where the figure of the father was mainly involved in leisure and play time, outings outside the home, or occasional supervision while the mother performs other care tasks.

For the fathers in the study, caring for their children with disabilities is a task they share with their mothers, to a greater or lesser extent. Their participation depends above all on the time that their jobs allow them to spend at home and on whether their wives contribute financially to the household’s upkeeping [17]; thus, those who work full-time devote less time to routine care and are more involved in occasional care [6,16]. Even so, fathers affirm that the time they are at home is also dedicated to these care routines (showering, feeding, and dressing). Previous studies show that the amount of time fathers spend with their children will be much greater when the mother is employed; in dual earner couples the division of childcare is often more evenly distributed between both parents.

According to the literature [6,17], mothers are the main caregivers of children with disabilities and those who usually leave their jobs to devote themselves to caring for their children, although in this study most of the mothers have had to leave their jobs, so that this fact is not disproved, it shows that fathers who are involved in raising a child with disabilities must also readjust their working hours or give them up if they want to actively participate in the care of their children. In some families, mothers are the breadwinners. Historically, care has always been understood as a female responsibility [4,17]. However, deciding how the birth of a child with a disability affects these parents has been debated in these couples. Aspects such as the “ease” of requesting a reduction in working hours or leave of absence, or access to a job if one was not previously available, how close the home was to work, or who had the best salary were taken into account, considering that reducing working hours or giving up work would leave the best possible economic conditions at home.

On the other hand, it has been observed that the time that one or the other devotes to care is always different, first, it depends on who works. The fathers interviewed did not mention other issues as a constraint to caring for their children on a full-time basis. For those fathers who are not full-time caregivers, the mornings before going to school, evenings after 7 o’clock and weekends are the times when they spend the most time with their children; they affirm that women spend twice or three times as much time as they do in caregiving. This is consistent with studies that show that fathers spend approximately 31 h per week caring for their children compared to the 50 h. per week that mothers spend.

For both parents taking on the birth of a child with a disability is a complicated event. Fathers described it as difficult to cope with, a time filled with fears and uncertainty. Although the literature suggested that guilt could be a problem for these fathers [16,18], most of the men interviewed did not describe this emotion. Some studies [18] state that stress is often more noticeable in mothers than fathers and sometimes this may be the case. However, the fathers in this study were quite emotionally affected after the birth of their children, some because of the stress of caregiving and others because they were not yet coming to terms with the reality of their children [16].

Consistent with some studies, these fathers admitted that they appeared strong to others, hiding their vulnerability, and acknowledged having high expectations regarding the development and course of their children, even comparing them to other children their age [16]. Anger, rage, or disappointment were not emotions described by the fathers in the study [18,19]. Tired, down, frustrated, nervous, down in the dumps, this is how they describe themselves most of the time. All the fathers seem to suffer more emotional than physical damage, some remember their process with a lot of bitterness, but they relate it more to their personality than to the fact of being a parent. Physically they do not relate their ailments to caring for their children but to age. On the contrary, they consider that caring for their children keeps them active. This is not coherent with what previous studies say, since when care falls to women, osteomuscular problems become noticeable [6,9]. Other studies suggest that the physical and emotional well-being of mothers is poorer compared to fathers [10], with mothers reporting muscle aches, high levels of anxiety and stress, burning out, physical fatigue, exhaustion, feelings of intense anger, guilt, and depression. Some of these symptoms coincide with those mentioned by the fathers in our study. This suggests further research to determine if there are significant differences between fathers and mothers who regularly care for their children with disabilities.

Contrary to the literature [18], most of the fathers in the study regularly attend the social, health and educational resources their child attends, they are also involved in their child’s treatment: they are trained and sometimes perform the physical therapies themselves at home. For most of the fathers, not being able to spend as much time as they would prefer at home is something that worries them when they work, they would prefer to share the caring tasks with their wives. The latter is consistent with what most participants in a previous study indicated [19], namely, that they would prefer to have more flexibility in their jobs to be able to participate in childcare, as they feel they are letting their family down by not being present. Having more flexibility would allow them to be more involved in medical appointments and treatments. For some fathers, quality time with their children, showing them affection and love, as well as being part of their daily care is extremely important, sometimes even more so than financially.

Caring leads many of the participants to neglect their self-care and reduce the number of hours devoted to their own needs, and to activities linked to personal satisfaction and leisure. Caring for their children implies putting aside enjoyment with their partner and friends, but this is something they do not relate directly to their children having a disability, but rather to the fact of being fathers. In addition, the incidence of marital separation and family disharmony is higher in families with children with disabilities. Separation and disharmony are more likely where there have been previous marital difficulties. In the case of this small sample of fathers, for those who were already close before the birth of their child, couples were able to maintain their relationships without allowing the stress of their child’s impairment to become a barrier to communication and caring. For others, however, the added stress has deteriorated it, sometimes to the point of divorce. It would be interesting to be able to track these relationships over time to identify whether stress over the long term and as the child grows older becomes a problem.

Among the limitations of this work, it is worth considering the exploratory nature of the study, given the enormous difficulties in finding a sufficiently large sample of fathers with minor children with disabilities who wanted to participate. The sample is also made up of fathers of children of different ages and disabilities. The interviews were all telephone interviews. Although this technique allowed for the inclusion of fathers from other areas of the country, it may have reduced the richness of the interviews due to the absence of visual observation. It is also important to bear in mind that, given the object of the study as they may understand it as “an examination” about their involvement in the care of their children, there was a possibility that participants may have been reluctant to give socially unacceptable answers, for fear of being judged. As a result, the findings cannot be generalized to other groups, but it opens the door to future lines of research.

A larger study, including a more diverse group and a larger number of participants could be useful and would allow this research to be generalized to a larger population. It would also be interesting to study whether increasing men’s involvement in caregiving decreases the stress and negative consequences normally associated with mothers.

## 5. Conclusions

Caring for a child with a disability has important consequences for the physical, mental, and social well-being of fathers, as it has a significant impact on different areas of their lives. However, their involvement in caregiving is always job dependent. Fathers of children with disabilities who are heavily involved in their care perceive disturbances in the work environment, neglect their social participation, lack time for leisure, for their partner, for sports or rest, in short, for taking care of themselves. Furthermore, the parents state that the news of the arrival of a child with a disability is something difficult to cope with and has an important impact on the couple.

In future research it would be relevant to contemplate the experiences of parents who live alone or who have children with other types of disabilities.

## Figures and Tables

**Table 1:** Characteristics of the participants.

Parent	P1	P2	P3	P4	P5	P6	P7
Age	44	36	39	49	46	46	33
No. of children	2	2 twins	2	2	2	2	1
Age of child with disability	4 y 14	1 year	5	12	14	13	6
Sex of child	Both boys	Boy and girl	Girl	Boy	Boy	Boy	Boy

## Supplementary Materials

The following are available online at https://www.mdpi.com/article/10.3390/healthcare10010014/s1, Table S1: interview script.
