# Supplementary material for: Fathers in the Care of Children with Disabilities: An Exploratory Qualitative Study
Source: Healthcare (Basel). 2021 Dec 22;10(1):14. doi: 10.3390/healthcare10010014 (PMC8775232; doi:10.3390/healthcare10010014)
Supplement: Supplementary file 1 [file healthcare-10-00014-s001.zip › healthcare-1370046-supplementary.pdf]

Table S1. Interview script.

- Age of parent and child. Disability of the child.
- How were you informed of your child's diagnosis? How long ago was it?
- How did you feel immediately after receiving the diagnosis? How do you think your wife felt? How do you feel now with your child's diagnosis? How does your wife feel?
- How do you think your child's condition limits him/her? What difficulties do you perceive?
- Does your child require constant care because of his/her disability? Why?
- Who is responsible for the regular care of your child? What do you take care of and how much time do you spend on it?
- What childcare tasks do you usually participate in? How much time do you spend on these tasks?
- If you work, how do you balance work and childcare?
- Who is in charge of showering, feeding or dressing him/her, if your child needs it? And who is in charge of playing with him/her, taking him/her to the park, doing homework...? Who attends hospitals and therapy sessions with the child?
- If any of them left the job after the diagnosis, how did they make the decision?
- How do you think the tasks you perform differ from those performed by your wife?
- Who else is involved in caring for your child? What are you responsible for?
- Do you think that having a child with a disability has changed your life? In what way? Have you stopped doing activities that you used to do?
- How has your child's diagnosis impacted your relationship?
- Have you noticed any changes in your physical, mental or emotional health since caring for your child?
